# 对苯二胺类橡胶防老剂及其醌类转化产物分析方法研究进展

**DOI:** 10.3724/SP.J.1123.2025.01017

**Published:** 2025-08-08

**Authors:** Jiawen CHENG, Jiashun ZHU, Yajing LIU, Jing HUA, Shuang LI

**Affiliations:** 1.双星集团有限责任公司，山东 青岛 266400; 1. Doublestar Group Co. ，Ltd. ，Qingdao 266400，China; 2.山东省青岛生态环境监测中心，山东 青岛 266003; 2. Qingdao Eco-environment Monitoring Center of Shandong Province，Qingdao 266003，China; 3.青岛科技大学高分子科学与工程学院，山东 青岛 266042; 3. School of Polymer Science and Engineering，Qingdao University of Science & Technology，Qingdao 266042，China; 4.青岛理工大学环境与市政工程学院，山东 青岛 266520; 4. School of Environmental and Municipal Engineering，Qingdao University of Technology，Qingdao 266520，China

**Keywords:** 对苯二胺, 橡胶防老剂, 样品前处理, 仪器检测, 分析方法, *p*-phenylenediamine, rubber antioxidants, sample preparation, instrumental analysis, analytical methods

## Abstract

对苯二胺类化合物（PPDs）以其优异的抗氧化和抗臭氧性能成为橡胶工业中重要的抗老化添加剂。但其在使用与释放过程中形成的醌类转化产物（PPD-Qs）具有潜在的环境风险与生态毒性。PPDs及PPD-Qs已在空气、水体、沉积物和生物体等多种介质中检出，且通常浓度低，反应活性高，易受复杂基质干扰，给准确定量带来挑战。本文系统梳理了PPDs及PPD-Qs在不同类型样品中的分析检测方法研究进展，涵盖气态、液态与固态等多种介质的样品前处理与检测策略。在气态样品中，滤膜采样与溶剂洗脱流程有助于目标物的有效富集与干扰去除；对于液态样品，固相萃取（SPE）技术显著提升PPDs及PPD-Qs的富集效率并降低基质效应；固态/半固态样品（如沉积物和海产品）的分析中，超声辅助萃取（UAE）结合净化策略则展现出对痕量目标物的优异萃取能力。仪器检测方面，色谱-质谱联用技术凭借其高分辨率和高灵敏度已成为主流手段，可实现PPDs及PPD-Qs的高效识别与准确定量；电化学传感技术则在快速响应与便携监测方面展现出良好的应用前景。本文综合评估了各类分析策略的适用性与技术优势，为PPDs类污染物的检测方法优化与应用提供了理论依据和技术支持。

## Abstract (en)

*p*-Phenylenediamine（PPD） compounds are widely used as antidegradants in the rubber industry due to their excellent antioxidant and antiozonant properties. However， increasing environmental concerns have arisen regarding their transformation products， especially quinone derivatives（PPD-Qs）， which are formed through oxidative processes under environmental conditions. These compounds have been frequently detected in various matrices， including air， water， sediment， soil， and biota， and have demonstrated significant ecological toxicity even at trace concentration. Accurate quantification of PPDs and PPD-Qs remains a significant analytical challenge due to their low environmental concentrations， high chemical reactivity， and matrix interferences. This review critically evaluates the current state-of-the-art analytical methodologies for the determination of PPDs and PPD-Qs across various environmental compartments. Emphasis is placed on the latest advancements in sample pretreatment techniques and instrumental detection methods that are suitable for complex and heterogeneous matrices. In gaseous and particulate samples， quartz fiber filters， passive samplers， and accelerated solvent extraction（ASE） have been employed for effective compound collection and extraction， with antioxidant protection（e.g.， glutathione） used to prevent analyte degradation. solid-phase extraction（SPE） based on hydrophilic-lipophilic balance（HLB） sorbents， as well as salting-out assisted liquid-liquid extraction（SALLE）， have achieved high recovery rates and reduced matrix effects. Passive monitoring approaches like diffusive gradients in thin films（DGT） have enabled long-term， time-integrated assessment of waterborne PPD-Qs under varying environmental conditions. For solid or semi-solid matrices such as soil， sediment， and biological tissues， ultrasound-assisted extraction（UAE）， gel permeation chromatography（GPC）， and modified QuEChERS methods have been widely adopted for high-efficiency extraction and purification. Instrumentally， gas chromatography-mass spectrometry（GC-MS） and liquid chromatography-tandem mass spectrometry（LC-MS/MS） remain the gold standards for sensitive and specific quantification. High-resolution mass spectrometry（HRMS）， such as Orbitrap platforms， enables non-targeted screening and structural elucidation of unknown metabolites and degradation products. In addition， emerging techniques such as condensed phase membrane introduction mass spectrometry（CP-MIMS） provide rapid， in situ detection with minimal sample pretreatment， showing great potential for real-time environmental monitoring. Electrochemical sensing platforms based on carbon-based or metal nitride-modified electrodes have also demonstrated promise due to their rapid response， cost-effectiveness， and field-deployable capabilities. However， challenges such as electrode fouling and selectivity limitations remain. In conclusion， this review integrates and evaluates a wide range of analytical approaches for detecting PPDs and PPD-Qs in complex environmental matrices. The comparative analysis of methodologies provides practical insights for optimizing analytical performance and advancing environmental surveillance. Future research should prioritize the development of automated， high-throughput， and green analytical platforms that are adaptable to field monitoring， risk assessment， and regulatory needs for emerging tire-derived contaminants.

对苯二胺类化合物（*p*-phenylenediamines， PPDs）是一类广泛应用于橡胶工业的防老剂，其通过捕捉自由基和抑制氧化链式反应^［1］^，有效延缓橡胶的老化和性能衰减，从而显著延长橡胶材料的使用寿命^［2］^。60%的橡胶用于轮胎的制备，因此，这使得PPDs在汽车轮胎等领域得到了广泛应用^［3-6］^。具有代表性的PPDs包括*N*-（1，3-二甲基丁基）-*N′*-苯基对苯二胺（*N*-（1，3-dimethylbutyl）-*N′*-phenyl-*p*-phenylenediamine， 6PPD）、*N*，*N*-二苯基对苯二胺（*N*，*N′*-diphenyl-*p*-phenylenediamine， DPPD）和*N*-异丙基-*N′*-苯基对苯二胺（*N*-（1，4-dimethylpentyl）-*N′*-phenylbenzene-1，4-diamine， IPPD）^［7］^。其中，6PPD凭借卓越的抗氧化能力、优异的环境适应性以及显著增强橡胶机械性能的能力^［8，9］^，成为PPDs家族中应用最广泛、关注度最高的防老剂。据统计，全球每年生产轮胎已超过31亿条^［10］^，对6PPD的需求量已超过20万吨^［11-13］^。

然而，作为橡胶工业中重要的防老剂，6PPD在其广泛应用的同时，不可避免地通过轮胎磨损颗粒的扩散、道路径流的冲刷以及废弃橡胶制品的不当处理进入环境^［14，15］^。研究表明，这类污染物可随水文输送沿河流迁移，并最终汇入海岸带和海洋沉积物，可能对沿海生态系统构成潜在风险^［16，17］^。在氧气、光照和氧化剂等条件下，释放至环境中的6PPD发生化学转化，生成其醌类转化产物——6PPD-Q^［18，19］^。6PPD-Q因高毒性成为环境和生态系统中的重要风险因子，使其被视为一类新污染物。研究发现，6PPD-Q能够在极低浓度（µg/L级别）下对水生生物产生显著毒害^［20］^。例如，北美水域发生的大规模银大马哈鱼（*Oncorhynchus kisutch*）死亡事件^［10］^，这一事件引发了全球范围内的高度关注。面对这一问题，多个地区相继采取严格的监管措施。美国华盛顿州5931号法案中明确要求全面审查和管控含有6PPD的机动车轮胎，美国环保署更是将6PPD-Q列为优先监测污染物，建议全面评估其环境暴露风险^［21］^。近年来，我国科研院所也逐步加强了对6PPD及其转化产物的关注，并在相关研究和污染监测方面取得进展^［22，23］^。这表明6PPD及6PPD-Q的环境行为和生态毒性已成为全球关注的热点问题。

在6PPD及6PPD-Q对环境和生态系统潜在危害的推动下，研究PPDs及其醌类转化产物（PPD-Qs）在环境介质中的分布、迁移和转化规律显得尤为重要^［24-27］^，因此，准确监测PPDs及PPD-Qs在不同基质中的含量和分布，对于理解其环境行为及生态影响至关重要。然而，这类化合物的定性定量分析仍面临诸多技术挑战。一方面，PPDs及PPD-Qs在环境中通常以痕量形式存在（ng/L或ng/kg级别）^［28-30］^，其检测结果易受复杂基质中有机质、无机离子及颗粒物干扰^［31，32］^。另一方面，这类化合物具有较高的化学活性和相似的分子结构，异构体或类似物在色谱分离中易发生峰重叠，从而限制了定性定量分析的准确性。

为应对PPDs及其转化产物分析中的技术挑战，研究者们致力于开发先进的样品前处理及仪器检测方法^［31-37］^，以提高其分析灵敏度和准确性。在此背景下，本文综述了近3年PPDs及PPD-Qs分析方法的研究进展，重点聚焦于色谱-质谱联用技术在复杂基质中痕量目标物检测的应用。此外，系统探讨了样品前处理技术的优化策略、现存局限性及发展趋势。本综述还展望了未来研究方向，包括开发高效分析方法、解决复杂基质干扰问题及实现多组分化合物的同时检测，旨在为新污染物的精准分析和风险评估提供理论支持。

## 1 PPDs及PPD-Qs的结构及理化性质

PPDs及其转化产物PPD-Qs的高效分析与其独特的分子结构和理化性质密切相关^［38］^。PPDs的分子结构以对苯二胺为核心，在氨基（-NH_2_）上引入不同的烷基或芳基取代基。芳胺基团的电子供体特性使其能够捕捉自由基并生成稳定的中间体，从而表现出优异的抗氧化性能^［39］^。同时，分子结构中取代基的类型对其功能特性具有重要影响。例如，6PPD中的长链烷基取代基不仅显著增强了其抗氧化效率，还改善了其在橡胶基质中的溶解性和分散性^［40］^；而芳基取代基则有助于进一步提高分子耐久性和化学稳定性^［41］^。这种结构功能的结合使PPDs在橡胶工业中得到广泛应用。

表1列出了几种常见PPDs及PPD-Qs的结构式与关键物化性质。

**表 1:** 常见PPDs及PPD-Qs的结构式及其物化性质

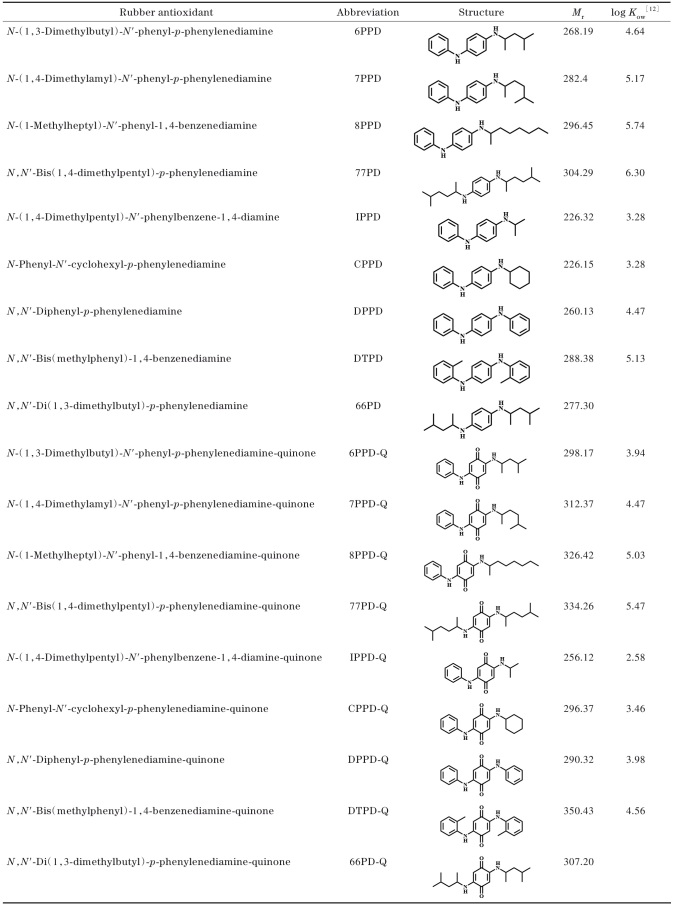

然而，PPDs的结构特性不仅决定了其在橡胶工业中的广泛应用，也使其在环境中具有显著的反应活性。在环境条件下，PPDs易发生氧化反应，生成其主要转化产物——PPD-Qs。与PPDs相比，PPD-Qs的分子结构发生了显著变化，从而改变了化学性质^［42］^。PPD-Qs的生成通常经历两个主要氧化步骤^［34，43］^：第一步，臭氧与芳香环发生亲电反应，形成羟基中间体；第二步，通过进一步的氧化，羟基转化为羰基，最终形成稳定的醌环结构^［44］^。这种结构转变赋予PPD-Qs更高的化学活性和氧化能力，使其与环境介质（如溶解性有机质或矿物表面）的相互作用潜力显著增加。此外，相较于母体PPDs，羰基的存在增加了PPD-Qs的极性，使其亲水性相对提高。这一变化在正辛醇-水分配系数（log *K*_ow_）的下降中有所体现，该值从母体PPDs的3.28~5.13降至2.58~4.56。这一理化性质的改变，使得PPD-Qs比PPDs更易通过地表径流、大气沉降等过程向下游水体输送，最终进入海洋环境^［45-47］^。这种复杂的迁移和转化行为对PPD-Qs的生态风险评估提出了更高的要求。因此，深入研究PPD-Qs的结构特性与环境行为对于开发高效分析方法、推动精准环境监测具有重要意义。

## 2 PPDs及PPD-Qs的样品前处理技术

PPDs及PPD-Qs复杂的化学特性和痕量水平的分布特性，使得样品前处理成为其分析中的关键步骤，直接决定了目标物的富集效率和分析结果的灵敏度及准确性。由于这类目标物广泛分布于气态、液态以及固态/半固态等复杂介质中，选择合适的样品前处理技术尤为重要。针对这些挑战，研究者们开发了多种创新性的样品前处理策略，以有效平衡目标物的富集效率和基质干扰去除效果。

### 2.1 气态样品

气态样品中PPDs及PPD-Qs分析需充分考虑其物化性质及基质的复杂性。这类化合物通常附着于大气颗粒物中，并具有较高的化学活性，易与有机杂质发生反应，增加了储存和分析的难度。因此，在气态样品的分析过程中，采样与前处理是两个关键环节，选择合适的采样方法，并优化后续的提取和净化步骤，对于提高检测的准确性和灵敏度至关重要。香港浸会大学蔡宗苇团队^［31］^开发了一种基于石英纤维滤膜的主动采样与前处理方法，用于高效捕集大气颗粒物中的PPDs及PPD-Qs。为克服基质干扰，该方法对滤膜进行550 ℃高温处理以去除有机杂质，采样后将滤膜冷冻保存（-20 ℃）以保持目标物的化学稳定性。样品采集完成后，通过超声辅助萃取（以二氯甲烷（dichloromethane， DCM）和乙腈（acetonitrile， ACN）作为萃取溶剂），结合氮吹浓缩的方式对样品进行净化和富集。实验结果表明，该方法在不同环境条件下具有良好的适用性，10种PPDs及PPD-Qs的回收率为74%~96%，检出浓度低至0.13 pg/m^3^，为低浓度PPDs及PPD-Qs的定量分析提供了可靠支持。

除了主动采样方法，被动采样凭借其无电力依赖、操作简便和低碳环保等优势，逐渐成为气态样品中目标物监测的重要方法^［58］^。加拿大学者^［49］^采用聚氨酯泡沫盘式采样器结合加速溶剂萃取（accelerated solvent extraction， ASE）技术，实现了全球特大城市空气中轮胎污染物的监测，检出限（limits of detection， LODs）低至0.169~2.71 pg/m^3^。这一方法展现了被动采样技术在PPDs及PPD-Qs环境监测中的潜在应用价值。此外，美国密西西比大学James Cizdziel团队^［59］^对Sigma-2被动采样器进行了改进，通过引入聚四氟乙烯阀门设计（图1），有效避免了采样过程中颗粒物的流失，同时结合高分辨轨道阱质谱技术，将LODs降至2.13~2.90 ng/L。该方法不仅提高了目标化合物的采集效率和检测灵敏度，还具有低碳、环保及操作简便的优势。

**图1:**
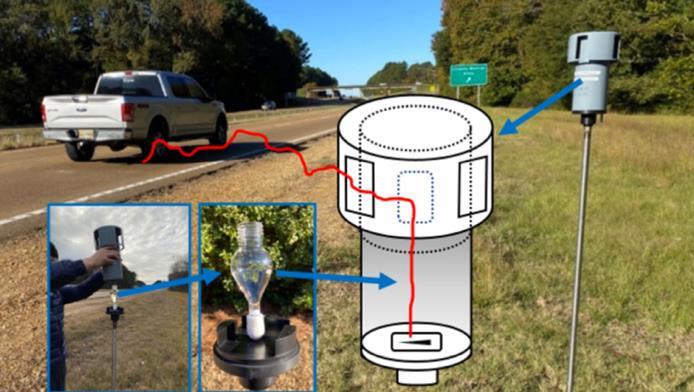
改进后的Sigma-2被动采样器示意图^［59］^

在气态样品的前处理过程中，目标物的化学稳定性同样需要关注。PPDs及其转化产物在氧气、光照及其他环境因素的作用下可能发生氧化降解，影响分析的准确性。为降低这一影响，中国科学院大学刘文彬团队^［51］^在分析粉尘样品时，为防止PPDs在样品制备过程中发生氧化降解，预先加入0.2 mL 1 mmol/L谷胱甘肽作为保护剂，该抗氧化策略显著提高了分析稳定性，9种PPDs及PPD-Qs的回收率达到71%~105%。

### 2.2 液态样品

与气态样品的分析类似，在液态样品的检测中，添加保护剂同样是提高分析准确性的重要策略。暨南大学杜碧柏等^［53］^基于盐析液液萃取（salting-out assisted liquid-liquid extraction， SALLE）技术，建立了尿液样品中6PPD及6PPD-Q的分析方法。该研究者在尿液样品中加入乙腈作为有机相，并添加谷胱甘肽作为保护剂，同时加入硫酸铵促进相分离。该方法的回收率为62%~65%，LODs为0.012~0.021 ng/mL。相比传统LLE，SALLE利用盐析作用增强了目标物的相分离效率，同时减少了有机溶剂的使用。然而，谷胱甘肽虽能减少6PPD氧化降解，但对其他潜在代谢产物的稳定性影响仍需进一步研究。

近年来，固相萃取（solid phase extraction， SPE）技术因其富集效率高、适用性广，成为液态样品前处理的主流技术^［60］^。加拿大萨斯喀彻温大学Markus Brinkmann团队^［52］^采用亲水亲脂平衡（hydrophilic lipophilic balance， HLB）SPE小柱处理雨水样品，该小柱的亲水亲脂平衡特性使其能够同时有效捕获雨水样品中的极性和非极性化合物，确保目标物回收率为70%~88%。同时，HLB小柱的高吸附容量能够实现6PPD-Q的高效富集分析（LOD为1.2 ng/mL），使得该SPE小柱成为处理液态样品的首选。另一方面，为了满足水环境中新污染物持续监测的需求，中国科学院烟台海岸带研究所陈令新等^［61］^开发了一种基于菲克第一扩散定律的新型原位被动采样装置——HLB-扩散梯度薄膜（diffusive gradients in thin films， DGT）。其中，扩散层通常由琼脂糖凝胶和过滤膜构成，负责控制目标物的扩散速率，并确保扩散过程的稳定性。当目标物通过扩散层到达结合层后，被结合层中的HLB选择性捕获。随着采样时间的延长，结合层中的目标物累积，装置表面与结合层界面之间形成稳定的线性浓度梯度（如图2所示）。这一设计使HLB-DGT能够在不同水体环境中实现PPDs及PPD-Qs的高效富集和长期稳定监测。通过系统优化，装置在不同复杂环境条件下（pH 6.5~8.5、离子强度0.000 1~0.5 mol/L、溶解性有机物质量浓度0~20 mg/L）均具有良好的稳定性和可靠性。结果表明，在河水和融雪样品中，6PPD-Q的检出质量浓度分别为15.8~39.5 ng/L和210 ng/L，尤其是融雪样品的持续监测结果显著高于传统抓取采样法的瞬时浓度结果。这一发现不仅表明融雪可能是6PPD-Q的重要运输途径，也证明了HLB-DGT装置在雪样中目标物持续释放监测中具有显著优势。

**图2:**
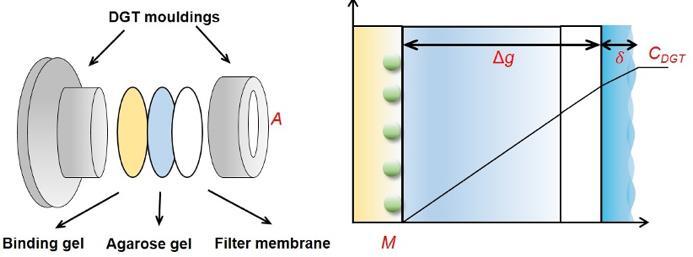
DGT装置结构与原理图^［61］^

### 2.3 固态/半固态样品

对于固态样品来说，土壤是最具代表性的基质之一，其样品前处理技术的优化研究为其他固态样品的分析提供重要借鉴。中国科学院大连化学物理研究所陈吉平等^［55］^采用超声辅助萃取（ultrasound-assisted extraction， UAE）结合凝胶渗透色谱净化（gel permeation chromatography， GPC）技术，建立了土壤样品中PPDs及PPD-Qs的分析方法。结果显示，该法中7种PPDs及PPD-Qs的LODs为0.24~2.29 ng/g，加标回收率为82.87%~100.57%，基质效应为88.29%~108.13%。通过结合UAE和GPC技术，该方法不仅提高了目标物的萃取效率，还显著减少了干扰物对分析结果的影响，为土壤样品中PPDs及PPD-Qs的精准检测提供了技术支撑。

暨南大学曾力希团队^［29］^采用UAE结合离心分离技术，建立了海洋沉积物样品中PPDs及PPD-Qs的高效分析方法。沉积物样品经过冻干和过筛处理后，加入谷胱甘肽作为保护剂。结果显示，该方法的回收率为81.2%~93.4%，相对标准偏差（relative standard deviation， RSD）小于10%，基质效应为82.7%~95.2%，没有基质增强或抑制的现象发生。本研究强调了在PPDs及PPD-Qs的分析中添加谷胱甘肽保护剂对抑制氧化过程的重要性，并展示了UAE技术在复杂沉积物样品处理中的强适用性，可推动海岸带沉积物中新污染物的精准监测。

针对食品样品分析受脂类和糖类等干扰物影响这一问题，中国农业大学马永强团队^［56］^开发了一种改良的QuEChERS方法用于蜂蜜和鱼样中6PPD及6PPD-Q的分析。该方法通过硅胶基伯胺仲胺键合相（primary secondary amine， PSA）与C18联合净化策略，利用PSA的氨基功能团有效去除样品中的极性干扰物（如有机酸、糖类和色素等）^［62］^，C18通过其非极性吸附特性吸附样品中的脂类和其他非极性干扰物，从而显著降低基质效应对检测的干扰。优化后的QuEChERS方法在鱼类和蜂蜜样品中对6PPD及6PPD-Q的回收率可达73.3%~108.3%，并将基质效应校正至70.4%~95.6%范围内，从而保证了检测的灵敏度和准确性。相比于使用单一吸附剂，PSA与C18的联合应用显著提升了净化效率，可更好地适配脂类和糖类含量较高的复杂基质，展现出优越的实用性。

尽管QuEChERS方法对食品样品的处理具有显著优势，但针对水产品这一更复杂基质，前处理技术仍需进一步创新。为此，上海农业科学院宋卫国等^［63］^开发了一种基于抗氧化剂保护的盐析辅助萃取（salt-out assisted extraction， SAE）结合多滤柱过滤净化（multi-plug filtration clean-up， mPFC）的样品前处理方法（如图3所示），用于分析水产品中的PPDs及PPD-Qs。该方法针对目标物在萃取过程中易因接触氧气和自由基（如单线态氧^1^O_2_、羟基自由基⋅OH和超氧阴离子O_2_^-^⋅）而发生光氧化或热氧化降解的问题^［22］^，创新性地在萃取过程中加入抗坏血酸和3，5-二叔丁基-4-羟基苯甲酸作为抗氧化剂。这些抗氧化剂通过捕获自由基和抑制氧化反应，有效减少了目标化合物的降解。结合m-PFC净化技术，该方法通过有效吸附脂类和其他干扰物，显著提升了分析性能，实现了PPDs及PPD-Qs的高灵敏度、高精密度（LOD为0.003 00~0.020 0 μg/kg，精密度<13.9%）分析。研究结果表明，该方法特别适用于鱼、虾和蟹类等复杂水产品的快速高效净化。

**图3:**
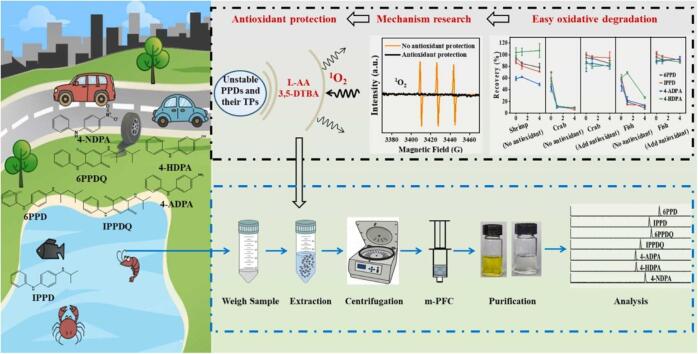
抗氧化剂保护-盐析辅助萃取-多滤柱过滤分析水产品中PPDs及PPD-Qs的流程图^［63］^

综上，近3年来应用于复杂基质中PPDs及PPD-Qs分析的主要样品前处理方法及其应用场景见表2。这些方法在不同类型样品中的应用价值日益突出。例如，SPE和DGT技术显著提高液态样品中痕量目标物的检测效率。UAE结合GPC技术能够有效降低固态样品的基质干扰，改良的QuEChERS方法和SAE技术则在食品及水产品样品处理中表现出卓越性能。然而，这些技术仍存在基质干扰以及操作繁琐性等问题，亟需通过创新进一步优化。未来的研究应着力于开发绿色低碳的样品前处理方法，设计多功能高选择性吸附材料，推动高通量自动化设备的应用，并构建集成多技术的平台，满足复杂基质中新污染物筛查与识别的需求。在此基础上，结合实时动态监测装置的研发，将进一步拓展对PPDs及其转化产物迁移行为的认知深度，为生态风险评估提供关键数据支撑。

**表 2:** 复杂基质中PPDs及PPD-Qs的样品前处理方法

Sample	Analytes	Extraction method	Extraction/elution solvent	LOD	Recovery/%	Ref.
Air	IPPD， DPPD， CPPD， 6PPD， DNPD， 77PD and 6PPD-Q	UAE	ACN， DCM/HEX（1∶1， v/v）	0.3-75 pg/mL	84-110	［^48^］
Air	IPPD， DPPD， CPPD， 6PPD， and 6PPD-Q	ASE	PE/ACE （83∶17， v/v）	0.169-2.71 pg/m^3^（LOQ）	-	［^49^］
Air particulate	IPPD， DPPD， CPPD， 6PPD， DTPD， 77PD， 7PPD， 8PPD， 66PD， IPPD-Q， DPPD-Q， CPPD-Q， 6PPD-Q， DTPD-Q， 77PD-Q， 7PPD-Q， 8PPD-Q and 66PD-Q	UAE	DCN and ACE	0.078-0.602 pg/m^3^	67-94	［^50^］
Atmospheric particles	IPPD， DPPD， CPPD， 6PPD， DTPD， IPPD-Q， DPPD-Q， CPPD-Q， 6PPD-Q and DTPD-Q	UAE	DCN and ACE	0.019-0.296 ng/mL（IQL）	74-96	［^31^］
Dust	IPPD， DPPD， CPPD， 6PPD， DNPD， IPPD-Q， DPPD-Q， CPPD-Q and 6PPD-Q	UAE	ACN， DCM/HEX（1∶1， v/v）	0.05-0.35 ng/g（LOQ）	71-105	［^51^］
Dust	IPPD， DPPD， CPPD， 6PPD， DNPD， and 77PD	UAE	ACN， DCN/HEX（1∶1， v/v）	0.11-2.35 ng/g（LOQ）	71-103	［^36^］
Water	6PPD-Q	SPE	MeOH and DCM	1.2 ng/mL	70-88	［^52^］
Urban runoff water	IPPD， DPPD， CPPD， 6PPD， DTPD， IPPD-Q， DPPD-Q， CPPD-Q， 6PPD-Q and DTPD-Q	SPE	MeOH/DCN （1∶9， v/v）	0.019-0.296 ng/mL（IQL）	73-93	［^31^］
Urine	6PPD and 6PPD-Q	SALLE	ACN	0.012-0.021 ng/mL	62-65	［^53^］
Urine	IPPD-Q， DPPD-Q， CPPD-Q， 6PPD-Q， and DTPD-Q	LLE	DCM and ammonia	2.4-7.5 pg/mL	80.5-114	［^54^］
Soil	IPPD， DPPD， CPPD， 6PPD， DTPD， IPPD-Q， DPPD-Q， CPPD-Q， 6PPD-Q and DTPD-Q	UAE	DCM and ACN	0.019-0.296 ng/mL（IQL）	70-113	［^31^］
Soil	IPPD， DPPD， CPPD， 6PPD， DTPD， 77PD， 7PPD， 8PPD， 66PD， IPPD-Q， DPPD-Q， CPPD-Q， 6PPD-Q， DTPD-Q， 77PD-Q， 7PPD-Q， 8PPD-Q and 66PD-Q	UAE	DCN and ACE	0.10-0.77 ng/g	64-105	［^50^］
Soil	IPPD， DPPD， CPPD， 6PPD， DNPD， 77PD and 6PPD-Q	UAE and GPC	UAE： ACE； GPC： DCM	0.24-2.29 ng/g	82.87-100.57	［^55^］
Honey and fish	6PPD and 6PPD-Q	QuEChERS	ACN	0.00025-0.0003 mg/kg	73.3-108.3	［^56^］
Zebrafish Embryos	IPPD， CPPD， 6PPD， and DTPD	UAE and PSA purified	DCM	0.38-0.68 ng/g	73.1-225	［^57^］
Tire tissue	IPPD， DPPD， CPPD， 6PPD， DTPD， 77PD， 7PPD， 8PPD， 66PD， IPPD-Q， DPPD-Q， CPPD-Q， 6PPD-Q， DTPD-Q， 77PD-Q， 7PPD-Q， 8PPD-Q and 66PD-Q	UAE	DCN and ACE	0.002-0.018 µg/g	72-117	［^50^］

ACN： acetonitrile； DCM： dichloromethane； HEX： hexane； PE： petroleum ether； ACE： acetone； MeOH： methanol； UAE： ultrasound-assisted extraction； ASE： accelerated solvent extraction； SPE： solid phase extraction； SALLE： salting-out assisted liquid-liquid extraction， LLE： liquid-liquid extraction； GPC： gel permeation chromatography； QuEChERS： quick， easy， cheap， effective， rugged， safe； PSA： primary secondary amine； LOD： limit of detection； IQL： instrumental quantification limit； LOQ： limit of quantification.

## 3 PPDs及其转化产物的仪器检测方法

PPDs及其转化产物PPD-Qs复杂的化学结构及多样化的分布特性使其检测成为分析科学领域的一个重要课题。随着研究的深入，针对PPDs及PPD-Qs的检测方法逐步发展出色谱法和电化学检测法等多维度、互补性强的技术体系，不仅为其分布、迁移和环境行为的研究提供了可靠的技术支撑，也为新污染物的筛查与识别奠定了技术基础。

### 3.1 气相色谱/气相色谱-质谱分析

6PPD和6PPD-Q是中等摩尔质量的有机化合物（摩尔质量分别为268.19 g/mol和298.17 g/mol），具有适当的挥发性和热稳定性，这使得它们在GC的高温进样过程中能够顺利气化，同时避免因热分解或降解而损失信号。

河南农业大学刘炳衫等^［64］^通过DB-WAX色谱柱分离橡胶样品中的防老剂，结合GC-火焰离子化检测器（flame ionization detection， FID）对6PPD进行定量分析。结果显示，该方法在分离能力和灵敏度方面表现优异，线性范围为0.01~1.5 mg/L，LOD为2.6 µg/L。尽管GC-FID在灵敏度上具有一定优势，但其定性能力仍存在局限性，尤其是在复杂基质中进行痕量化合物分析时，难以准确识别目标化合物的结构。为克服这一局限，广东工业大学陈智锋等^［57］^采用GC-MS法分析斑马鱼胚胎中的PPDs，该法采用电子轰击电离（70 eV）和选择反应监测模式，实现了超痕量PPDs的高灵敏检测（线性范围为0.1~200 µg/L，相关系数大于0.99，LODs为0.38~0.68 ng/g）。通过该方法成功检测出鱼类胚胎样品中4种PPDs，为这类物质的生物暴露研究提供了详实的数据。

尽管GC和GC-MS技术具有卓越的分离和检测性能，但其对目标物的热稳定性要求较高，因此在处理热敏性化合物或复杂基质时，可能会受到一定限制。

### 3.2 液相色谱/液相色谱-质谱分析

LC因无需高温处理，成为PPDs及PPD-Qs检测的另一重要技术手段。河南农业大学测试中心团队^［64］^采用C18色谱柱，并在295 nm波长下对6PPD进行测定。结果表明，该方法的线性范围为0.02~10 mg/L，检出限和定量限分别为6.0 µg/L和20.0 µg/L，回收率为98.47%~103.28%。尽管LC法操作简便且成本较低，但在复杂基质中其定性能力存在局限，难以解析目标化合物的详细结构。

为解决这一问题，LC-MS，特别是高效液相色谱-轨道阱高分辨质谱（HPLC-Orbitrap MS）成为应对复杂基质分析的主流工具。中山大学杨欣团队^［34］^采用HPLC-Orbitrap MS，研究了水中6PPD在模拟日光的条件下向6PPD-Q转化的过程。该方法采用正离子模式的电喷雾电离，精确解析了中间体6PPD-OH（［M+H］^+^， *m/z* 285.196 6，误差为1.8×10^-6^（1.8 ppm））和6PPD-（OH）_2_（［M+H］^+^， *m/z* 301.1905，误差为2.0×10^-6^（2.0 ppm））的分子结构及转化路径（图4），为研究这类化合物的分布、归趋及环境风险评估提供了强大技术手段。

**图4:**
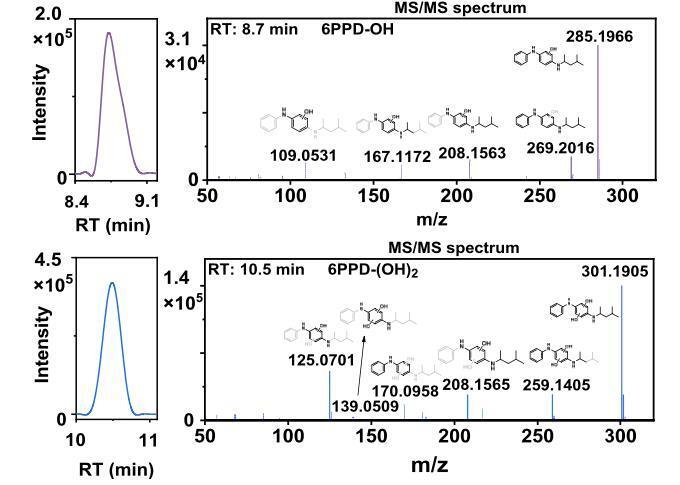
水中的6PPD经模拟日光照射后6PPD-OH和6PPD-（OH）_2_的检出结果^［34］^

同样地，香港浸会大学蔡宗苇团队^［65］^采用高分辨率Orbitrap-MS结合数据依赖采集（data dependent acquisition， DDA）模式和LC-MS/MS对PM_2.5_样品中的化合物进行了筛查和结构确认。在广州和太原的PM_2.5_样品中，检出了*m/z* 335.269 3的分子离子峰，其色谱保留时间（18.39 min）与合成标准物完全匹配。再通过二级碎片离子信息确认，*m/z* 237.1595为C₇H₁₅基团的碎片离子，*m/z* 139.050 2为苯胺骨架碎片，最终确认该物质为77PD-Q，这是首次在PM_2.5_中检出该化合物。通过这一筛查方法，成功识别到另外6种PPD-Qs及8种PPDs，并进一步验证其在环境空气中的普遍存在性。为了进一步解析PPD-Qs的来源，团队采用Spearman相关性分析和回归模型，评估PPD-Qs是否由其相应的PPDs转化而来。结果表明，PPD-Qs的环境浓度与其对应PPDs具有相关性，其中6PPD-Q与6PPD、77PD-Q与77PD具有显著相关性，分别为0.53~0.84（*p*<0.000 1）和0.91~0.96（*p*<0.000 1），表明这些大气环境中的醌类衍生物很可能由其对应的PPDs氧化形成的。此外，为了研究6PPD生物转化路径，暨南大学曾力希等^［53］^采用靶向分析的方法，对6PPD的体外代谢产物进行定性与定量分析。首先，利用HRMS比对6PPD及其已知代谢产物（如6PPD-Q），确定其特征碎片离子并建立定量方法。在此基础上，该团队在人肝微粒体体外代谢实验中，对6PPD的降解进行时间分辨监测。结果表明，6PPD在3 h内降解了65%，但6PPD-Q的生成量极低，仅占总代谢的不到2%，这一现象表明6PPD在体内并不主要转化为6PPD-Q，而是可能沿着其他代谢途径降解。该研究通过体外代谢实验结合基于LC-MS/MS的尿液生物监测，进一步证实人体尿液中的高浓度6PPD-Q主要来源于环境氧化，而非生物代谢，为6PPD的健康风险评估提供了重要数据支持。

### 3.3 直接质谱分析

近年来，随着对6PPD及6PPD-Q在水体中高频次、低浓度、复杂基质背景下的监测需求快速提升，传统LC-MS/MS在前处理耗时、样品通量和响应速度等方面的瓶颈日益凸显。对此，Krogh团队^［66］^开发了一种基于凝聚相膜引入质谱（condensed phase membrane introduction mass spectrometry， CP-MIMS）的直接检测策略，将亲疏水选择性膜采样技术与串联质谱系统耦合，通过将中空纤维（polydimethylsiloxane， PDMS）膜探头直接浸入环境水样，实现6PPD及6PPD-Q的实时富集与直接离子化检测，无需任何前处理步骤。该方法不仅显著缩短了分析时间（单样本检测周期可压缩至2.5 min），满足高通量筛查需求，同时具备低LOD（8 ng/L），已覆盖银大马哈鱼等水生生物的毒性响应浓度区间（6PPD-Q对幼年银大马哈鱼的中位致死浓度LC_50_为95 ng/L），具有良好的生态预警潜力。在实际应用中，其对复杂天然水体亦显示出较强的适应性，无需萃取、净化或色谱分离，即可完成有效检测。此外，该方法兼具原位响应和在线监测能力，可用于动态过程中的污染物释放行为追踪，如轮胎颗粒中6PPD及其氧化产物的实时迁移评估。总体而言，该研究提出了一种“前处理-检测一体化”的新型分析方法，为环境新污染物监测技术从传统实验室分析向原位、动态感知的方向转变提供了新路径。

### 3.4 电化学检测法分析

电化学传感（electrical conductivity， EC）技术以其低成本、便携性和高灵敏度的特点，在PPDs及PPD-Qs检测方面展现了重要的应用潜力。美国芝加哥洛约拉大学Christophe Renault等^［67］^通过循环伏安法和差分脉冲伏安法结合玻碳电极，实现了对6PPD的高灵敏检测（LOD低至10 nmol/L）。其高灵敏度主要得益于6PPD分子中的苯环结构与电极表面石墨的*π-π*相互作用（吸附常数达1.2 L/µmol），促使其在电极表面形成致密单分子层。此外，6PPD在电化学检测中表现出的可逆氧化还原特性，具有典型的两电子、两质子的转移机制，其特征峰在不同pH条件下具有线性漂移特性（每pH单位变化59 mV），展现了该电化学反应的可控性。然而，该法在实际应用中面临着一些挑战。当6PPD浓度超过4.6 μmol/L时，电极表面过度吸附导致活性位点饱和，不仅显著降低灵敏度和效率（检测时间延长至1 h），还可能引发电极表面的阻塞效应，影响电化学反应的动力学过程，从而导致信号不稳定和方法重现性下降。在6PPD的电化学传感检测最新研究中，郑州大学谢正坤团队^［68］^开发了一种基于氮碳介导的*γ*-Mo₂N纳米复合材料电化学传感器，用于快速检测环境中的6PPD。该传感器采用改性碳糊电极（carbon paste electrode， CPE），展现出高稳定性和良好的电子传输性能，LOD为4.48 ng/mL。研究表明，该复合材料的增强碱性促进了6PPD的去质子化，材料的（200）晶面在6PPD的解离中起到关键作用，从而提高了传感器的响应能力。此外，该传感器在对不同地区土壤样品中的6PPD检测方面表现出良好的抗干扰能力，可为环境污染物实时评估和监测提供可靠的技术支持。

综上所述，针对PPDs及PPD-Qs的检测方法已经发展出多维度、互补性强的技术体系。其中，色谱-质谱法作为经典的分离技术，通过结合多种高灵敏度检测器，在复杂环境样品和生物样品的痕量分析中展现出卓越的灵敏度和定性定量能力，为PPDs及PPD-Qs的分布和环境归趋研究提供了可靠的数据支撑。而电化学检测法则以快速响应和便携性为特色，凭借低成本、易操作的优势，以及创新的电极材料开发，不断提升灵敏度和选择性，进一步拓展其在现场检测中的应用潜力。表3总结了现有前处理和仪器检测方法的主要优势与局限，为新污染物的筛查、污染溯源及生态风险评估提供理论依据。

**表3:** PPDs及PPD-Qs的样品前处理及仪器检测方法优缺点总结

Pretreatment/ detection technique	Advantages	Disadvantages
LLE	simple， versatile， effective for large sample volumes and low equipment cost	high solvent consumption， emulsification issues， incomplete phase separation， time-consuming and limited selectivity
SALLE	enhanced phase separation， lower organic solvent consumption， improved recovery for hydrophilic compounds and reduced risk of emulsion formation	limited applicability to non-polar compounds， potential analyte loss due to salt interactions， optimized salt selection required and limited compatibility with certain organic solvents
SPE	enhanced selectivity and purification， higher concentration and sensitivity， automation compatibility and time efficiency	limited sample capacity， complex method optimization and risk of cartridge blockage
UAE	simple， faster extraction time， lower solvent consumption and broad applicability to various matrices	potential degradation of sensitive compounds， limited selectivity and interference from solid particles
GPC	efficient molecular size separation， minimal analyte interaction and high reproducibility and precision	high solvent consumption， limited compatibility with highly polar or ionic compounds and time-consuming
ASE	higher extraction efficiency， significant time savings， automation and high throughput	high initial equipment cost， limited suitability for heat-sensitive compounds and potential for co-extraction of undesired compounds
QuEChERS	minimal equipment requirement， simple and rapid procedure， minimal sample preparation required and high matrix compatibility	limited selectivity， not suitable for all analytes， inconsistent performance for highly lipophilic compounds and need for additional sample cleanup in protein-rich or highly pigmented samples
GC-MS	strong separation capability， capable of analyzing thermally stable and non-polar compounds	limited to volatile and thermally stable compounds， derivatization may be required and limited ability to analyze aqueous samples directly
LC-MS	compatible with complex and biological samples， wide range of ionization techniques and capable of analyzing large biomolecules	matrix effects and ion suppression， solvent and additive compatibility limitations， not ideal for highly volatile compounds
HRMS	high mass accuracy and resolution， superior identification of unknown compounds and versatile ionization and coupling options	high instrumentation cost， complex data processing and interpretation， limited availability of reference databases and challenges in ion suppression and matrix effects
EC	simple and rapid analysis， non-destructive testing， cost-effective and low maintenance	interference from multiple ion types，requires calibration and maintenance，limited sensitivity for low-ionic-strength samples and electrode fouling in certain applications

## 4 结论与展望

目前，PPDs及其醌类转化产物PPD-Qs的分析检测技术在复杂基质中取得了重要进展。总体来看，LLE和SALLE操作简便，成本较低，但选择性有限；SPE和ASE提高了富集效率和回收率，但前者需关注填料是否过载，后者设备成本较高；UAE、GPC及QuEChERS技术适用于复杂基质，但部分方法选择性不足。在检测仪器方面，GC-MS可用于PPDs的检测，但对高极性转化产物的适用性可能较差，而LC-MS能覆盖不同极性的PPDs及其转化产物，成为主流检测手段。HRMS在解析PPDs氧化及环境转化机制方面展现出独特优势，但数据处理复杂，非靶向筛查仍需优化。此外，EC技术虽具备便携、快速检测能力，但对PPDs及其醌类产物的专一性识别能力仍需提升。

PPDs及其转化产物的环境分析研究已从初步识别走向机制解析与高效监测阶段。未来研究亟需聚焦其在多种环境介质（水、土壤、大气）中的赋存特征，尤其在人类活动密集区域（如城市道路、工业园区及排水管网）中，其释放强度大、迁移路径复杂、生态风险不容忽视，并且需要在技术集成化、绿色化和智能化方向上实现突破^［69，70］^，发展适应不同介质、具备现场响应能力的分析方法体系，推动污染暴发早期预警、风险阈值识别及区域污染溯源等实际应用，以全面应对复杂环境中新污染物筛查与监测的需求。
